# Clinical Evaluation of Two Non-Invasive Genetic Tests for Detection and Monitoring of Urothelial Carcinoma: Validation of UroVysion and Xpert Bladder Cancer Detection Test

**DOI:** 10.3389/fgene.2022.839598

**Published:** 2022-06-06

**Authors:** Niko Kavcic, Ivan Peric, Andreja Zagorac, Nadja Kokalj Vokac

**Affiliations:** ^1^ Department of Urology, University Medical Centre Maribor, Maribor, Slovenia; ^2^ Laboratory of Medical Genetics, University Medical Centre Maribor, Maribor, Slovenia

**Keywords:** urothelial carcinoma, bladder cancer (BC), urinary markers, UroVysion®, Xpert, detection, monitoring

## Abstract

A variety of commercially available urinary molecular markers have been introduced for detecting and monitoring urothelial carcinoma (UC). We prospectively evaluated the UroVysion^TM^ Bladder Cancer Kit (FISH) and the Xpert® Bladder Cancer Detection (Xpert) test. Both tests were performed on voided urine samples after negative cystoscopy and negative abdominal ultrasound (US) and/or negative computed tomography urography (CTU). Urine specimens from 156 patients diagnosed with hematuria and suspected of having UC and 48 patients followed up after treatment of UC were analyzed using FISH and Xpert. Among 204 patients, 20 had UC, 11 located in the bladder, six in the ureter, and three in the renal pelvis. FISH had an overall sensitivity (SN) of 78%, a specificity (SP) of 93%, and a negative predictive value (NPV) of 96%. Xpert had an overall SN of 90%, an SP of 85%, and an NPV of 98%. Both tests had high SN, SP, and NPV. The SP of FISH was significantly higher. By using FISH and Xpert in addition to cystoscopy, renal and bladder US, and/or CTU in the diagnostic workup of patients with hematuria and follow-up after transurethral resection of the bladder (TURB), a substantial number of patients (10%) otherwise missed were discovered to have UC.

## Introduction

Urothelial carcinomas (UCs) are the sixth most common tumors in developed countries. They can be located in the lower and/or the upper urinary tract. Bladder tumors represent 90–95% of UC (Babjuk et al., 2017). Hematuria is the most common finding in non-muscle-invasive bladder cancer (NMIBC). Visible hematuria was found to be associated with a higher stage of disease than nonvisible hematuria (Ramirez et al., 2016). The most common symptom of upper urinary tract urothelial cell carcinoma (UUTUC) is visible or nonvisible hematuria (70–80%) (Inman et al., 2009; Cowan, 2012). The prevalence of bladder cancer (BC) in patients with microhematuria is only 1% in referred populations (Ashley N. Gonzalez et al., 2019). Patients with hematuria and patients during follow-up after treatment for UC are advised to perform voided urine cytology, cystoscopy, renal and bladder ultrasound, and/or computed tomography urography (CTU). The use of diagnostic flexible ureteroscopy (FURS) and biopsy is recommended if imaging and cytology are not sufficient for the diagnosis and/or risk-stratification of the tumor (Rouprêt et al., 2021). The examination of voided urine for exfoliated cancer cells has high sensitivity in G3 and high-grade tumors (84%), but low sensitivity in G1/LG tumors (16%) (Yafi et al., 2015). The sensitivity in carcinoma *in situ* (CIS) detection is 28–100% (Têtu 2009). Cytological interpretation is user-dependent (Raitanen et al., 2002).

Because of the low sensitivity of urine cytology, urinary molecular marker tests have been introduced for detecting and monitoring UC. The UroVysion^TM^ Bladder Cancer Kit (UroVysion Kit) is approved by the U.S. Food and Drug Administration (FDA) and designed to detect aneuploidy for chromosomes 3, 7, and 17 and loss of the 9p21 locus *via* fluorescence *in situ* hybridization (FISH) in urine specimens. Almost 20 years have passed since UroVysion was approved by the FDA (Hajdinjak 2008; Nagai et al., 2021). Results from the UroVysion Kit are intended for use in conjunction with current standard diagnostic procedures, as an aid for the initial diagnosis of bladder carcinoma in patients with hematuria and subsequent monitoring for tumor recurrence in patients previously diagnosed with bladder cancer.

Xpert® Bladder Cancer Detection (Xpert; CE-IVD, Cepheid, Sunnyvale, CA, United States) quantitates the expression of five mRNA targets that may be overexpressed in BC (Wallace et al., 2018). It is an easy-to-use urinary test with improved SN and NPV compared with cytology and UroVysion. It represents a promising tool for identifying hematuria patients with a low likelihood of BC (Franciscus Johannes P. van Valenberg et al., 2021). A similar test (Xpert Bladder Cancer Monitor) has been validated in the surveillance setting for monitoring BC patients (F. Johannes P. van Valenberg et al., 2019).

In this prospective study, we evaluated and compared the performance of UroVysion FISH and Xpert in the detection of UC in patients with hematuria and in the monitoring of UC after TURB.

## Materials and Methods

### Patients

In the described study, approved by the Ethics Commission (UKC-MB-KME No. 24-09/17), 204 patients were enrolled, followed by signed informed consent. In total, 156 patients were suspected of having UC because they had previously been diagnosed with hematuria, and 48 patients were monitored for tumor recurrence as they were previously diagnosed with UC. The exclusion criteria were a history of urinary stone disease, ongoing urinary tract infection, or an invasive procedure of the urinary tract in the past 3 months. Subjects with hematuria were defined as those with gross or microscopic hematuria. Voided urine specimens were collected a few days after white light cystoscopy. The same sample of each patient was divided into two parts, one for FISH and one for the mRNA test. Both tests were performed on voided urine samples after negative cystoscopy and negative abdominal ultrasound (US) and/or computed tomography urography (CTU). Patients were enrolled from June 2017 to December 2020 with at least 6 months of follow-up. The frequency of follow-up cystoscopies and upper urinary tract imaging was based on the current EAU guidelines. Bladder cancer was diagnosed with biopsy, photodynamic cystoscopy, TURB procedures, and cystectomy procedures. UUTUC was diagnosed with URS/FURS procedures, biopsy of visible lesions, and nephroureterectomy procedures. The histopathological report on the transurethral resection of a bladder lesion was performed by the Histopathological Laboratory of the Department of Pathology of the University Medical Centre in Maribor, Slovenia. Tumors were evaluated according to the 2017 TNM classification of urinary bladder cancer (Paner et al., 2018) and graded according to the 2004/2016 WHO grade classification (Humphrey et al., 2016).

The sensitivity (SN), specificity (SP), and negative predictive value (NPV) of Xpert BC and UroVysion FISH were calculated and compared with final histology results.

### Fluorescence *In Situ* Hybridization (FISH)

Chromosomal alterations were detected using the UroVysion^TM^ test (Abbott Molecular, Inc., Des Plaines, IL, United States) which is a four-color FISH assay designed for the detection and quantification of chromosomes 3, 7, and 17 and the 9p21 locus on urine specimens fixed on slides. Voided urine was mixed with the preservative Carbowax (2% polyethylene glycol in 50% ethanol) 2:1 (v:v). Slide preparation and the test were performed according to the manufacturer’s instructions. The criteria for detecting bladder cancer by UroVysion are: ≥4 urothelial cells with a gain of ≥2 chromosomes 3, 7, or 17 or ≥12 cells with a loss of both copies of the 9p21 locus. In addition, >10 urothelial cells showing a gain for a single chromosome 3, 7, or 17 or >10 cells with tetrasomy or near tetrasomy for all chromosomes are also considered abnormal. A minimum of 25 morphologically abnormal cells were analyzed. Cells showing either a gain of multiple chromosomes (i.e., 3 or more signals) for more than one of the probes CEP 3 (red), CEP 7 (green), or CEP 17 (aqua) or a homozygous loss of locus 9p21 (gold) (i.e., no signals for LSI 9p21) were recorded. Each sample was analyzed until either ≥4 cells with gains of multiple chromosomes or ≥12 cells with homozygous loss of 9p21 were detected or until the entire slide was analyzed. Results were reported as positive, negative, or no cells (if the criterion of a minimum of 25 morphologically abnormal cells was not met) (Bollmann et al., 2005; Zellweger et al., 2006; Halling and Kipp 2008; Dimashkieh et al., 2013; Ye et al., 2014).

### mRNA-Based Urine Test

For measuring the levels of five target mRNAs (ABL1, CRH, IGF2, UPK1B, and ANXA10) by a reverse transcriptase polymerase chain reaction (RT-PCR), urine samples were analyzed using the Xpert® Bladder Cancer Detection test (Cepheid, Sunnyvale, CA, United States), according to the manufacturer’s protocol. A volume of 4.5 ml of voided urine sample was transferred to the urine transport reagent tube (Xpert Urine Transport Reagent Kit, Cepheid), and subsequently, 4 ml of pretreated urine was transferred to the reagent cartridge. All reagents needed for sample preparation, and RT-PCR were present in the self-contained reagent cartridge. Automated processing included capturing cells on a filter, lysis of cells by sonication, the addition of nucleic acid to dry the RT-PCR reagents, transfer to the reaction chamber, multiplex RT-PCR, and detection. ABL1 served as a sample adequacy control of human cells, and the ABL1 signal is required for a valid test result. Before the start of the PCR, the GeneXpert Instrument System measures the fluorescence signal from the probes to monitor bead rehydration, reaction tube filling in the cartridge, probe integrity, and dye stability. A “Cepheid internal control” (CIC), designed to detect sample-associated inhibition of the real-time RT-PCR, was included in each cartridge. Xpert Bladder Cancer Detection provides a “positive” or “negative” result based on the results of linear discriminant analysis (LDA) algorithm, which uses the cycle threshold (Ct) results of the five-target mRNA. A positive result is achieved when the LDA total (the result of an algorithm that uses the Ct values of ABL1, ANXA10, UPK1B, CRH, and IGF2) is equal to or above the cut-off point, the LDA total must be within the valid range of −20 to 20, ABL1 Ct must be within the valid range, and sample passes the probe check control. Not all mRNA targets need to be elevated for a positive test result. A negative result is achieved if the LDA total is below the cut-off point and the ABL1 Ct is within the valid range. The manufacturer determined the cut-off point of the LDA total at 0.4450 on the basis of statistical analysis of a large number of samples (Wallace et al., 2018; F. Johannes P.; van Valenberg et al., 2019; Pichler et al., 2018; Smrkolj et al., 2020). The result is ‘invalid’ if the presence or absence of target mRNAs cannot be determined, if the ABL1 Ct and/or CIC Ct do not meet the criteria, and if the cell content in the urine sample is too low or the PCR reaction was inhibited.

### Statistical Analysis

The diagnostic accuracy of the UroVysion FISH and Xpert Bladder Cancer detection tests was calculated, including SN, SP, and NPV. Both tests were assessed for the outcome of histologically proven UC (Paner et al., 2018). Data were analyzed using the SPSS software (Statistical Package for the Social Sciences, version 20.0, IBM Corp., Armonk, NY, United States) using the chi-squared test and t-test. The diagnostic value of the Xpert BC and UroVysion FISH was tested by determining the sensitivity (number of true positive tests/sum of a number of true positive and false negative tests), specificity (number of true negative tests/sum of true negative and false positive tests), and negative predictive value (number of true negative tests/sum of true negative and false negative tests). Sensitivity and specificity were compared using McNemar’s test. Receiver-operating characteristic (ROC) curves were plotted and the area under the ROC Curve (AUC) was calculated together with 95% confidence intervals (CIs). A *p*-value of less than 0.05 was considered statistically significant.

## Results

The study included 204 patients with a mean age of 63.1 ± 11.5 (SD) years, and 101 (49.5%) patients were male. Table 1 presents the test characteristics for the UroVysion FISH test and the Xpert BC Detection test.

**TABLE 1:** Comparison of the FISH (UroVysion® test) and Xpert (Xpert® BC Detection test) results.

	Xpert-invalid	Xpert-negative	Xpert-positive	All
FISH-no Cells	2	51	6	59
FISH-negative	2	104	17	123
FISH-positive	0	1	21	22
All	4	156	44	204

For 59 (29%) patients, we did not get a FISH result due to a lack of cells after harvesting the urine suspension prior to the FISH analysis, and for 4 patients (2%) we did not get the Xpert result due to an invalid attempt. Among those, 2 patients did not get the result with either of the tests, 21 patients had a positive result in both tests, and 104 patients had a negative result in both tests; 51 patients with no FISH result were Xpert-negative, and 6 patients with no FISH result were Xpert-positive; 2 patients with an invalid Xpert were FISH-negative, and 17 FISH-negative patients were Xpert-positive; 1 FISH-positive patient was Xpert-negative.

Among 145 FISH results, 4 out of 123 (3%) were false-negative and 8 out of 22 (36%) were false-positive and among 200 Xpert results, 2 out of 156 (1.3%) were false-negative and 26 out of 44 (59%) were false-positive.

Among 204 patients we detected 20 (9.8%) malignant tumors (Table 2): 11 bladder cancers, 6 ureter cancers, and 3 renal pelvis cancers. Of these, 6 were of low grade (LG): PUNLM (Papillary Urothelial Neoplasm of Low Malignant Potential) (*n* = 2), Ta (*n* = 4). The remaining 14 tumors were of high-grade (HG) CIS (*n* = 2), Ta (*n* = 1), T1 (*n* = 2), and ≥T2 (*n* = 9). Seven tumors were detected in a group of patients with hematuria and 13 in patients previously diagnosed with UC. Fifteen tumors were detected in men and 5 in women. The mean age of patients with tumors was 69.8 and 62.4 years for negative patients. The mean time from FISH and Xpert tests until the diagnosis of the malignant tumor was 10.3 months. Also, 13 tumors were detected with both tests, one was missed with both tests, 5 were detected with Xpert only, and one with FISH only.

**TABLE 2:** Sex, diagnosis, FISH, Xpert, cytology, imaging modality [US (ultrasound) and CT (computed tomography)], tumor location, and histology [a staging of tumor: PUNLM (papillary urothelial neoplasm of low malignant potential), CIS (carcinoma *in situ*), and Ta, T1, and ≥T2; HG = high grade; LG = low grade].

Patient	Sex	Diagnosis	FISH	Xpert	Cytology	Imaging modality	Tumor location	Hystology^a^
1	Male	Hematuria	Negative	Positive	Negative	US	Bladder	PULNM
2	Male	Previously UC	Positive	Positive	Negative	CT	Bladder	CIS
3	Male	Previously UC	Positive	Positive	Negative	US	Ureter	≥T2 LG
4	Male	Hematuria	Negative	Positive	Negative	CT	Bladder	Ta LG
5	Female	Previously UC	Positive	Positive	Atypia	US	Bladder	≥T2 HG
6	Male	Previously UC	Negative	Negative	Negative	US	Bladder	≥T2 HG
7	Male	Previously UC	Negative	Positive	Negative	CT	Ureter	Ta LG
8	Female	Previously UC	Positive	Positive	Atypia	US	Renal pelvis	≥T2 HG
9	Male	Hematuria	Positive	Positive	Suspicious	CT	Bladder	T1 HG
10	Male	Hematuria	Positive	Positive	Suspicious	CT	Ureter	≥T2 HG
11	Male	Hematuria	No cells	Positive	Suspicious	CT	Renal pelvis	≥T2 HG
12	Male	Previously UC	Positive	Positive	Negative	CT	Ureter	Ta LG
13	Male	Hematuria	Positive	Positive	Atypia	US	Bladder	PUNLMP
14	Male	Previously UC	Positive	Positive	Positive	US	Bladder	T1 HG
15	Female	Previously UC	No cells	Positive	Atypia	US	Ureter	Ta HG
16	Male	Previously UC	Positive	Negative	Positive	CT	Bladder	CIS
17	Female	Previously UC	Positive	Positive	Positive	US	Renal pelvis	≥T2 HG
18	Male	Hematuria	Positive	Positive	Positive	US	Ureter	≥T2 HG
19	Female	Previously UC	Positive	Positive	Negative	CT	Bladder	Ta LG
20	Male	Previously UC	Positive	Positive	Atypia	US	Bladder	≥T2 HG

All tumors were proven/validated with cytology/cystoscopy, upper urinary tract imaging, and confirmed histologically (Table 2).

FISH had an overall SN and SP of 78% (95% Cl: 52–93) and 93% (95% Cl: 88–97), respectively, and an NPP of 96% (95% Cl: 92–99) (Table 3). SN was 67% in hematuria patients and 83% in the previously UC group of patients; SP was 95% in hematuria patients and 86% in previously UC group of patients; and NPP was 98% in hematuria patients and 92% in previously UC group of patients.

**TABLE 3:** Sensitivity, specificity, and negative predictive value of FISH and Xpert tests.

	FISH	Xpert	—
Sensitivity			
All	78%	90%	*p* = 0.68
Hematuria	67%	100%	—
Previously UC	83%	85%	—
Specificity			
All	93%	85%	*p* = 0.004
Hematuria	95%	90%	—
Previously UC	86%	68%	—
Negative predictive value			
All	96%	98%	—
Hematuria	98%	100%	—
Previously UC	92%	92%	—

Xpert had an overall SN and SP of 90% (95% Cl: 68–98) and 85% (95% Cl: 80–90), respectively, and an NPP of 98% (95% Cl: 95–99) (Table 3). SN was 100% in hematuria patients and 85% in previously UC group of patients; SP was 90% in hematuria patients and 68% in previously UC group of patients; and NPP was 100% in hematuria patients and 92% in previously UC group of patients.

McNemar’s test showed that the overall SN (78 vs. 90%; *p* = 0.68) of the Xpert test was not significantly higher than that of UroVysion FISH. UroVysion FISH had significantly higher overall SP (93 vs. 85%; *p* = 0.004) than Xpert (Table 3).

**FIGURE 1:**
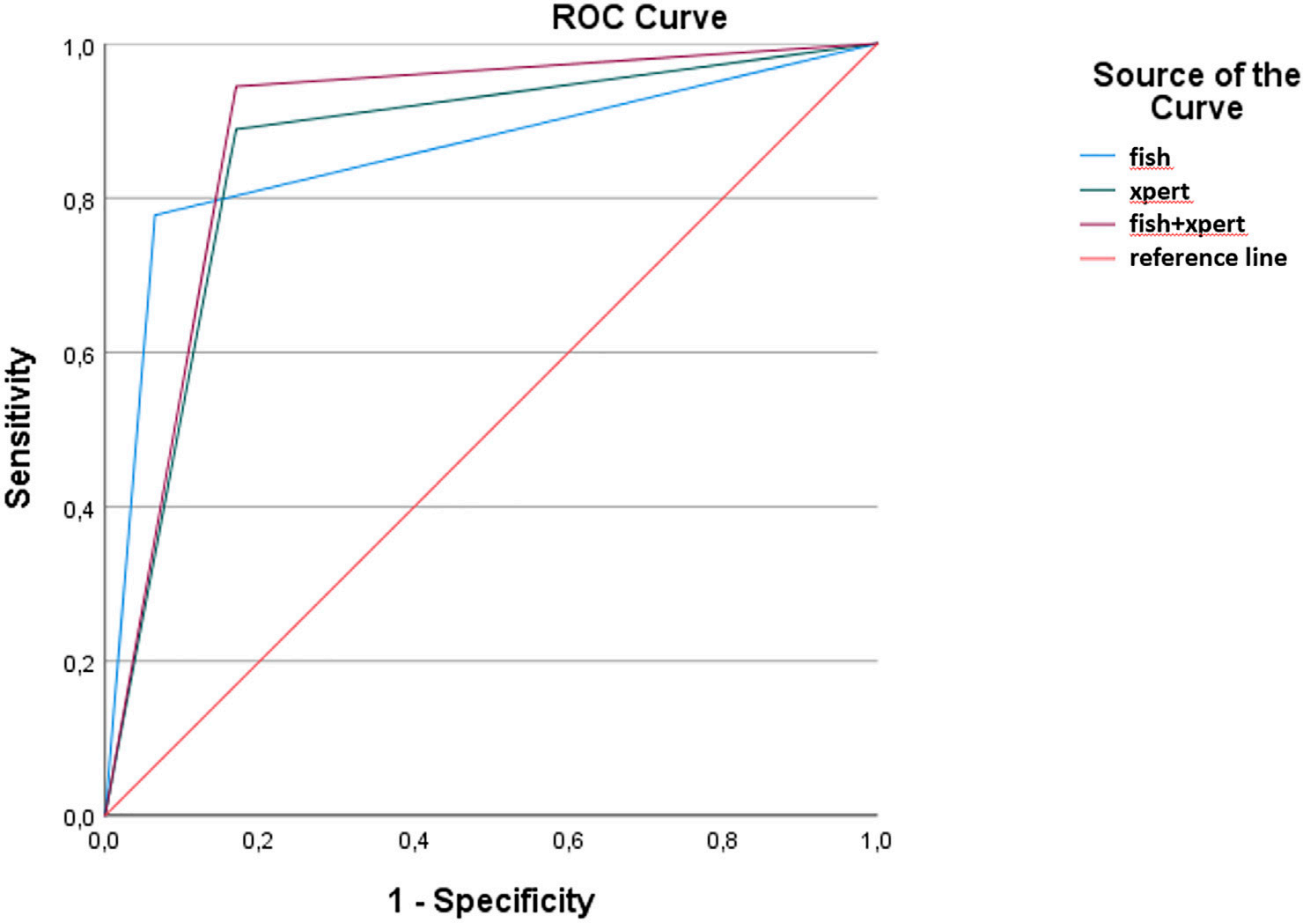
Receiver operating characteristic curves and areas under the curve (AUCs), including 95% CIs, were calculated for FISH, Xpert, and the combination of FISH and Xpert.

The ROC curve analysis (Figure 1) showed no difference between the Xpert (AUC = 0.86 and 95% CI 0.80–0.90; *p* < 0.001) and the FISH test (AUC = 0.85 and 95% CI 0.74–0.97; *p* < 0.001). Our data showed a slightly higher diagnostic accuracy (AUC = 0.89 and 95% CI 0.81–0.96; *p* < 0.001) when combining the Xpert and the FISH test.

## Discussion

The purpose of the present study was to evaluate two non-invasive tests that can be performed on voided urine in terms of screening patients with bladder cancer.

In our study, we showed that by using the UroVysion FISH test and the Xpert BC test in addition to cystoscopy, renal and bladder US, and/or CTU in the diagnostic workup of patients with hematuria and follow-up after TURB, a substantial number of patients otherwise missed were discovered to have UC. 10% (20 out of 204) of our patients had negative (unsuspicious for UC) cystoscopy, negative renal and bladder US, and/or negative CTU but were diagnosed with UC because of additional diagnostic procedures (biopsy) triggered by a positive UroVysion FISH and/or Xpert. The percentage of missed patients is in accordance with published data. The use of enhanced cystoscopy has highlighted the fact that white-light cystoscopy can miss up to 10–30% of cancers, especially carcinoma *in situ*, but also lower-grade disease (Svatek et al., 2005; Burger et al., 2013; Kang et al., 2017). Per-patient sensitivity of CTU for detecting upper urinary tract urothelial carcinoma (UUTUC) was estimated to be 93.5% (Jinzaki et al., 2011).

In our study, cytology was not included in the research protocol but was performed in most patients. In general, cytology has a high sensitivity for high-grade tumors but is limited by its low sensitivity (16%) for low-grade tumors (Siegel et al., 2013; Pichler et al., 2018). Even when using the Paris classification system, the sensitivity of urinary cytology for detecting low-grade non-muscle invasive bladder cancer (NMIBC) was low, ranging from 21 to 53% in an inter-observer variability analysis (McCroskey et al., 2015). In a meta-analysis of 56 studies with 22,260 patients, Mowatt et al. (2010) reported an SN of 44% (95% CI: 38–51%) and an SP of 96% (95% CI: 94–98%) for cytology. When cytology and Xpert results were combined in a multivariate analysis, the detection rate did not increase, indicating that cytology did not identify additional positive cases (Franciscus Johannes P. van Valenberg et al., 2021).

By comparing the UroVysion FISH test and the Xpert BC test, we showed high values of NPV for both tests. The NPV of FISH was 96% overall, 98% for the detection of UC in patients with hematuria, and 92% for monitoring after TURB. Similarly, the NPV of Xpert was 98% overall, 100% for detection, and 92% for monitoring. We observed higher values of SN for Xpert, 90% overall, 100% for detection, and 85% for monitoring and lower for FISH, 78% overall, 67% for detection, and 83% for monitoring. The difference in SN between FISH and Xpert was not significant. We observed higher values of SP for FISH, 93% overall, 95% for detection, and 86% for monitoring. Xpert had a SP of 85% overall, 90% for detection, and 68% for monitoring. Our data showed that FISH had a significantly higher overall SP (93 vs. 85%; *p* = 0.004) than Xpert. Our results are in line with systematic reviews and meta-analyses demonstrating an SN of 65–75% with a SP of 70% for UroVysion in the diagnosis of BC (Mowatt et al., 2010). A meta-analysis conducted on 2477 FISH tests showed an overall sensitivity of 72%, and a specificity of 83% (Hajdinjak 2008). In the study by Gomella et al. (2017), FISH was evaluated in the diagnosis of bladder and UUTUC. They concluded that FISH testing does offer a significantly higher detection of UC specifically for BC than voided cytology. For Gene Xpert Bladder Cancer Assay, Wallace et al. (2018) reported a sensitivity of 73% at an example cut-off point of 0.4 with 90% specificity in the hematuria population, 77% in the surveillance population, and 98% in the healthy and other controls. In the setting of detection of bladder cancer in patients with hematuria, Franciscus Johannes P. van Valenberg et al. (2021) reported that Xpert had an SN of 78% overall and 90% for high-grade tumors. The NPV was 98% overall. The SP was 84%. F. Johannes P. van Valenberg et al. (2019) studied patients under surveillance for bladder cancer and showed that Xpert had an overall SN of 74 and 83% for high-grade tumors. The NPV was 93% overall and 98% for high-grade tumors. The specificity was 80%. The Xpert SN and NPV were superior to those of cytology and UroVysion. In the study by Pichler et al. (2018), the overall sensitivity (84%) and NPV (93%) of the Xpert BC Monitor were significantly superior to those of bladder washing cytology (0.33 and 0.76; *p* < 0.001). B. Cowan et al. (2021) compared the performance of the Xpert Bladder Cancer Monitor, FISH, and cytology as a predictor of tumor recurrence. They found an overall sensitivity of 59, 45, and 23% for each test, respectively. They also showed that patients with a positive Xpert assay and negative cystoscopy were 2.7 times more likely to have a recurrence than patients with a negative Xpert and a negative cystoscopy result. The hazard ratio for experiencing a high-grade recurrence in the group with positive Xpert and a negative cystoscopy result was 6.8. It has to be taken into consideration that a positive urine marker in the setting of normal cystoscopy, US, and/or CTU could identify cancer before it can be detected visually (Seideman et al., 2015; Sharma et al., 2021).

In a high proportion of our samples (29% or 59 samples out of 204), no cells were found, so it was impossible to perform the FISH. 10% of those cell-free samples (six samples) were Xpert-positive, and 3% (two patients) had positive histology. Xpert was invalid in just 2% (four out of 204) cases. In our experience, Xpert was much easier and faster to perform and was conclusive in more samples than FISH. When FISH is negative, a sufficient number of cells need to be evaluated to exclude the presence of positive cells, so the process is observer-dependent.

In our study, we observed a higher than anticipated number of UUTUC cases, since UUTUCs are uncommon and account for only 5–10% of UCs (Siegel et al., 2013). Also, 45% of patients in our study (nine out of 20) were found to have UUTUC. The reason for such an observation could be patient selection, since only the patients with a negative cystoscopy and a negative abdominal US and/or a computed CTU were included. In such patients, UUTUC could be missed more easily than BC. According to EAU Guidelines (Rouprêt et al., 2021), the sensitivity of FISH for molecular abnormality characteristics of UUTUCs is approximately 50%; therefore, its use in clinical practice remains unproven (Chen and Grasso 2008; Johannes et al., 2010; McHale et al., 2019). Gomella et al. (2017) concluded that FISH does not appear to improve detection of urothelial carcinoma in patients with either UUTUC only or both BC and UUTUC. Fernández et al. (2012) examined the utility of UroVysion to detect UUTUC in the follow-up of patients after cystectomy and concluded it was not suitable. On the other hand, some studies have investigated the role of FISH in diagnosing UUTUC and reported promising results (Mian et al., 2010; Gruschwitz et al., 2014; Reynolds et al., 2014). Marín-Aguilera et al. (2007) showed that a FISH test performed on exfoliated cells from voided urine specimens has a greater sensitivity than cytology for detecting UUTUC while maintaining a similar specificity. Data on the performance of Xpert in the detection of UUTUC are lacking. Our results indicate that FISH and Xpert might have a role in identifying patients with UUTUC; however, future research is required to clarify the value of novel urinary markers in the detection of UUTUC.

By using the UroVysion Bladder Cancer Kit and the Xpert® Bladder Cancer Detection test in addition to cystoscopy, renal and bladder ultrasound, and/or computed tomography urography in the diagnostic workup of patients with hematuria and follow-up after transurethral resection of the bladder, a substantial number of patients otherwise missed were discovered to have urothelial carcinoma.

Both the UroVysion FISH test and the Xpert BC test had a high sensitivity, specificity, and negative predictive value.

In the pilot study presented, different genetic biomarkers were used to detect patients with UC, as the contribution to the development of bladder cancer depends on different genetic changes. Based on the small sample of patients analyzed, we can nevertheless suggest an algorithm for screening UC patients. In our opinion, it would be appropriate for each patient to have an Xpert analysis after cytology, and in the case of a positive result, to be tested with the FISH. The Xpert method is quick, easy, and cheaper compared to the FISH method, as the result is obtained within a few hours after urine collection, the result rarely falls out, and the procedure is not labor intensive. The FISH often has too little cellular material available for analysis and the result is not obtained, contrary to the Xpert. In this way, more potentially at-risk patients are captured.

Because the Xpert test has only been available for a few years, data on the performance of Xpert in the detection of UC are lacking. Our results indicate that FISH and Xpert might have a role in identifying patients with UC; however, future research is required to clarify the value of novel urinary markers in the detection of UC. We believe that our experience of using both tests on the same sample of patients is a useful contribution to the evaluation of screening tests using genetic biomarkers in urology.

## Data Availability

The original contributions presented in the study are included in the article further inquiries can be directed to the corresponding author.
